# Biochemical and structural characterization of polyphosphate kinase 2 from the intracellular pathogen *Francisella tularensis*

**DOI:** 10.1042/BSR20150203

**Published:** 2016-02-10

**Authors:** Laura E. Batten, Alice E. Parnell, Neil J. Wells, Amber L. Murch, Petra C. F. Oyston, Peter L. Roach

**Affiliations:** * Department of Chemistry, University of Southampton, Southampton, SO17 1BJ, U.K.; † Defence Science and Technology Laboratory, Porton Down, Salisbury, SP4 OJQ, U.K.; ‡ Institute for Life Sciences, University of Southampton, Southampton, SO17 1BJ, U.K.

**Keywords:** antibiotic sensitivity, enzyme kinetics, *Francisella tularensis*, kinase, polyphosphate, X-ray crystallography

## Abstract

The metabolism of polyphosphate is important for the virulence of a wide range of pathogenic bacteria and the enzymes of polyphosphate metabolism have been proposed as an anti-bacterial target. In the intracellular pathogen *Francisella tularensis*, the product of the gene FTT1564 has been identified as a polyphosphate kinase from the polyphosphate kinase 2 (PPK2) family. The isogenic deletion mutant was defective for intracellular growth in macrophages and was attenuated in mice, indicating an important role for polyphosphate in the virulence of *Francisella*. Herein, we report the biochemical and structural characterization of *F. tularensis* polyphosphate kinase (*Ft*PPK2) with a view to characterizing the enzyme as a novel target for inhibitors. Using an HPLC-based activity assay, the substrate specificity of *Ft*PPK2 was found to include purine but not pyrimidine nts. The activity was also measured using ^31^P-NMR. *Ft*PPK2 has been crystallized and the structure determined to 2.23 Å (1 Å=0.1 nm) resolution. The structure consists of a six-stranded parallel β-sheet surrounded by 12 α-helices, with a high degree of similarity to other members of the PPK2 family and the thymidylate kinase superfamily. Residues proposed to be important for substrate binding and catalysis have been identified in the structure, including a lid-loop and the conserved Walker A and B motifs. The ΔFTT1564 strain showed significantly increased sensitivity to a range of antibiotics in a manner independent of the mode of action of the antibiotic. This combination of biochemical, structural and microbiological data provide a sound foundation for future studies targeting the development of PPK2 small molecule inhibitors.

## Abstract (precis)

The polyphosphate kinase 2 (PPK2) from the intracellular pathogen *Francisella tularensis* has been characterized by a range of biochemical methods and X-ray crystallography. The antibiotic sensitivity of a deletion mutant lacking the gene encoding PPK2 is also reported.

## INTRODUCTION

Polyphosphate is an inorganic polymer which may extend to hundreds of phosphate residues linked as phospho-anhydrides and has been reported in cells from every domain of life [1]. At one time, polyphosphate was regarded as a ‘molecular fossil’ [2], providing a prebiotic source of phosphoanhydride equivalents [2] and without a precise described function, but recent discoveries have made it increasingly clear than nothing could be further from the truth. In eukaryotes, polyphosphate has been identified in a range of subcellular organelles [3] and has regulatory roles in a wide range of processes [4–7]. Polyphosphate is ubiquitous in prokaryotes [8], contributing to metabolic regulation during growth and development and contributing to the regulatory network that controls the response to stress and nutrient starvation [9–13]. The importance of polyphosphate to bacterial survival [8,9,14] and, in particular, it's correlation to the virulence of a wide spectrum of pathogens [14–17], has led to the proposal that enzymes from polyphosphate metabolism are potential therapeutic targets for anti-bacterial chemotherapy [14,18].

**Scheme 1:**
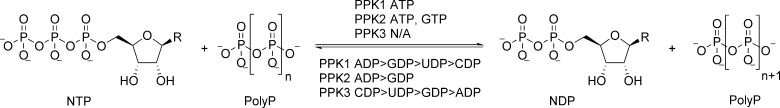
Reaction scheme for the three classes of polyphosphate kinases, PPK1, PPK2 and PPK3, indicating observed substrate specificity of the different enzyme classes

**Figure 1:**
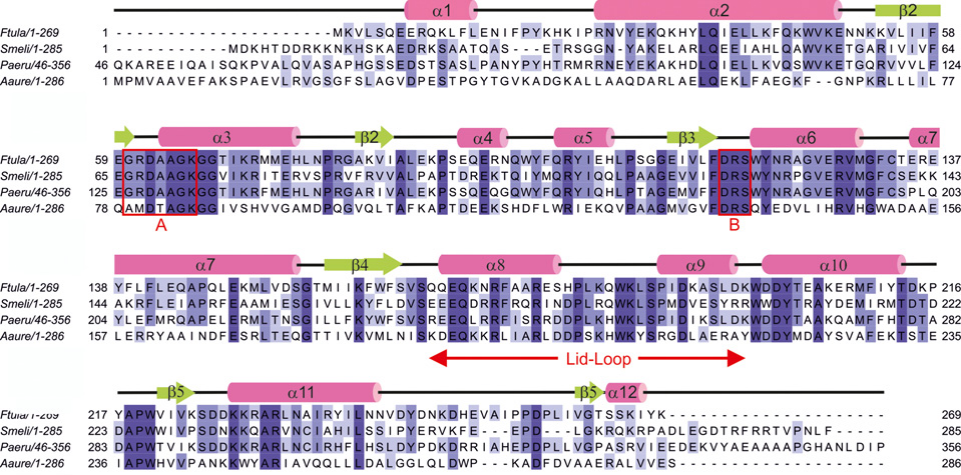
Alignment of PPK2 sequences from *F. tularensis* (labelled *Ftula*, accession code YP_170487.1, PDB code 4YEG), *P. aeruginosa* (*Paeru*, NP_248831, 3CZP), *S. meliloti* (*Smeli*, NP_384613, 3CZQ) and *A. aurescens* (*Aaure*, YP_949739.1, 3RHF) Residues are coloured by degree of conservation. Elements of secondary structure are taken from the *F. tularensis* structure (α-helices in pink, β-sheets in green). The Walker A and B motifs of PPK2 identified by Nocek et al. [23] are boxed in red. The unaligned N-terminal 45 residues of *Pa*PPK2 are omitted.

For the assembly of polyphosphate chains, three subclasses of bacterial polyphosphate kinases have been identified (Scheme 1). The polyphosphate kinase 1 (PPK1) family is found in the majority of bacterial species [19] and constitutes the principal polyphosphate biosynthetic enzyme in many bacteria [20,21] using ATP as the preferred phosphate donor. Sequence similarity identified a subclass of polyphosphate kinases which shows a preference towards pyrimidine nts [22]. Examples of polyphosphate kinase 2 (PPK2) have been characterized from a wide range of bacteria [18,23–26], where the kinetics favour polyphosphate-driven ATP synthesis. The 3D-fold of PPK2 (PDB codes 3CZP, 3CZQ and 3RHF) [23] is structurally distinct from PPK1 (PDB codes 1XDP and 1XDO) [27] and belongs to a larger family of P-loop kinases [28] that feature two conserved motifs that co-ordinate the nucleoside triphosphate and Mg^2+^, Walker A (GXXXXGK) and Walker B [23]. The Walker B motif was originally identified as hhhhD, where h represents a hydrophobic residue [29] and the aspartic acid is proposed to co-ordinate a magnesium ion. Earlier, the same aspartate in a DRS tripeptide conserved within the PPK2 family has also been termed the Walker B motif (Figure 1) [23]. The hydrolytic degradation of polyphosphate is catalysed by polyphosphatase (Ppx), resulting in the release of P_i_. As part of a regulatory network, Ppx is inhibited by (p)ppGpp which increases during the stringent response [8,14], resulting in the accumulation of higher levels of polyphosphate.

*Francisella tularensis* is an intracellular pathogen and the causative agent of the zoonotic disease tularemia [30]. It is noted for a low infectious dose, ease of dissemination and ability to cause severe disease [31]. These properties and the associated potential as a bio-threat led to the classification of *F. tularensis* as category A (the highest priority for prevention) by the Centers for Disease Control and Prevention (CDC) [32]. The availability of genome sequence data and development of molecular tools has allowed us to start to understand the molecular basis of *F. tularensis* pathogenicity. Bacteria may have one or more polyphosphate kinase homologues within their genomes [8] and some encode multiple polyphosphate kinase genes [23]. In *F. tularensis* subspecies *tularensis* SCHU S4, the gene FTT1564 and the homologue in *Francisella novicida*, FTN1472, were identified as encoding polyphosphate kinases of the PPK2 family (Figure 1). Inactivation of FTN1472 resulted in abolition of polyphosphate production, confirming the observation from bioinformatics analysis that this is the only polyphosphate kinase present in these strains [17].

Herein, we describe the biochemical characterization of the *F. tularensis* PPK2 encoded by FTT1564 (*Ft*PPK2), including activity assays using HPLC detection and ^31^P-NMR to measure product formation. These assays permitted the determination of the substrate preference and kinetic parameters of *Ft*PPK2. We report the affinity of *Ft*PPK2 for polyphosphate measured by isothermal titration calorimetry (ITC). We also report the crystal structure of *Ft*PPK2 and compare it with other enzymes of the P-loop phosphotransferase superfamily, identifying the conserved structural features and probable substrate-binding sites. In addition, we report the antibiotic sensitivity of the ΔFTT1564 strain. Taken together, these studies are an important prerequisite to investigating the development of *Ft*PPK2 inhibitors as potential anti-microbials.

## MATERIALS AND METHODS

### Materials

NMPs, NDPs, NTPs and DMHA were purchased from Sigma–Aldrich; DTT, BSA and antibiotics were purchased from Melford Laboratories; polyacrylamide/bis polyacrylamide (30% w/v, 37.5:1) and Bacto agar were purchased from Fisher Scientific; Bacto tryptone and yeast extract for culture media were purchased from Oxoid; Chelating Fast Flow resins were purchased from GE Healthcare; primers were purchased from Eurofins; BIO-X-ACT was purchased from Bioline; restriction enzymes and *Escherichia coli* strain K12 JM109 were purchased from New England Biolabs; *E. coli* BL21 Rosetta pLysS (DE3), the pET16b plasmid and polyphosphate averaging 25 units in length was purchased from Merck Chemicals. *E coli* strain TOP10 was purchased from Invitrogen. Screens (96-well), crystal trays and coverslips were purchased from Molecular Dimensions. Unless otherwise stated, other chemicals and reagents were and purchased from Sigma–Aldrich or Fisher Scientific.

### Protein expression and purification

The gene encoding *Ft*PPK2, FTT1564, was amplified from *F. tularensis* subspecies *tularensis* SCHU S4 genomic DNA using a forward primer (5′-gcggacatgttgcatcatcatc-atcatcataaagttttaagtcaagaagagcgc) paired with a reverse primer (5′-cgcctcgagttatttatatatttttgaagaagtgcctacgat). The PCR product was digested with PciI and XhoI and ligated into the NcoI/XhoI restricted pET16b. The resultant plasmid, pET16b/ppk, was verified by sequencing. The plasmid was chemically transformed into BL21 Rosetta pLysS (DE3). Single colonies were used to inoculate 2YT medium (10 ml containing 100 μg/ml ampicillin) and cultured overnight at 27°C. The overnight culture was used as a 1% inoculum into flasks of 2YT medium (500 ml) which was induced with IPTG (final concentration 0.4 mM) when the *D*_600_ reached 0.6 and then cultured overnight at 27°C. Cells were harvested by centrifugation (11,300 g for 20 min) and the cell pellet (typically ∼7 g/l of culture) was stored at–80°C. To purify *Ft*PPK2, the frozen cell pellet (∼35 g) was resuspended in cold lysis buffer (3 × w/v cell pellet) and a Roche protease inhibitor tablet added. Lysozyme (5–15 mg) was added and the cell suspension was stirred (4°C, 30 min), then sonicated on ice (4°C, 20×10 s with 10-s rest). The lysate was cleared by centrifugation (Sorval evolution, SLA-1500 rotor, 4°C, 29752 ***g***, 30 min) and the resulting supernatant was applied (4 ml·min^−1^) to a Ni-NTA Sepharose Fast Flow column (50 ml bed volume). The column was washed (4 ml·min^−1^) with low imidazole buffer (∼5 column volumes, 50 mM Tris/HCl, 0.5 M NaCl, 50 mM imidazole, 20% glycerol, pH 8.0). The *Ft*PPK2 was eluted with a gradient of 0%–100% high imidazole buffer (4 column volumes, 50 mM Tris/HCl; 0.5 M NaCl; 500 mM imidazole; 20% glycerol, pH 8.0). Fractions containing *Ft*PPK2 were pooled, dialysed (2×1 l, 50 mM Tris/HCl; 0.3 M NaCl; 20% glycerol; 5 mM DTT, pH 8.0) and stored at–80°C. For crystallization screening, *Ft*PPK2 was concentrated to 15 mg/ml in an Amicon pressure cell and applied to a S75 Sepharose gel filtration column (bed volume 200 ml, flow rate 2 ml·min^−1^). The eluted *Ft*PPK2 was eluted (2 ml·min^−1^; 50 mM Tris/HCl, 0.3 M NaCl, 20% glycerol, 5 mM DTT, pH 8) and fractions judged to be pure by SDS/PAGE analysis were pooled, concentrated to 15 mg/ml and stored as aliquots (100 μl) at–80°C.

### Ion-pair HPLC of *Ft*PPK2 activity assays

Substrate specificity of *Ft*PPK2 was analysed using an ion-pairing HPLC-based method. Reaction mixtures (1 ml) contained 50 mM Tris/HCl (pH 8.0), 0.3 M NaCl, 20% glycerol, 10 mM MgCl_2_ and 80 mM (NH_4_)_2_SO_4,_ 100 μM polyphosphate (as polymer), 0–2 mM nt and were initiated by the addition of *Ft*PPK2 (250 nM). Reactions were incubated at 37°C and at selected time points, aliquots (100 μl) were withdrawn and the reaction quenched by heating (95°C, 5 min). Precipitated protein was removed by centrifugation (13,200 g, 5 min) and a sample (40 μl) was analysed by ion pairing HPLC [Gemini C18 column (150×4.6 mm 5 micron)] with detection at 260 nm using the following solvents: organic, 80% methanol, 15 mM DMHA, pH 7.0, aqueous; 5% methanol, 15 mM DMHA, pH 7.0. The solvents were adjusted to pH 7.0 with acetic acid. The column was equilibrated in 25% organic at a flow rate of 0.8 ml·min^−1.^ After sample injection, the following elution profile was applied: 5 min isocratic (25% organic) and then a 22 minute linear gradient to 55% organic, followed by a 5 minutes isocratic at 55% organic, 5 min gradient to 25% organic and 5 min isocratic at 25% organic. Absorbance measurements were converted into concentrations of NTPs and NDPs using calibration curves and plotted as reaction time courses. For competition assays, the formation of product nts were empirically fitted to a single exponential rise to a maximum, [P]=[P]_max_(1–e^−^*^kt^*). Using the rate constant *k*, the initial rate of product formation, *v*, was calculated (*v*=*k*[P]_max_). For steady state kinetics, the time course of product formation was fitted to a linear function to give the initial rates which were then fitted to a classical Michaelis–Menten kinetic model.

### Metal ion and pH dependence of *Ft*PPK2

Substrate specificity of *Ft*PPK2 was analysed using an ion-pairing HPLC-based method. Reaction mixtures (500 μl) contained 50 mM Tris/HCl (pH 8.0), 0.3 M NaCl, 20% glycerol and 80 mM (NH_4_)_2_SO_4,_ 30 μM polyphosphate, 0.2 mM ADP and were initiated by the addition of *Ft*PPK2 (30 nM). MnCl_2_ and MgCl_2_ concentrations from 0.25 to 50 mM and a pH range from 5.5 to 9 were used, MES (50 mM) for the pH 5.5–6 and bis-tris propane (50 mM) for pH 6.5–9. Reactions were incubated at 30°C and, after 3 min, aliquots (100 μl) were withdrawn and the reaction quenched using EDTA (50 mM), followed by heating (95°C, 5 min). Precipitated protein was removed by centrifugation (13,200 g, 5 min) and a sample (40 μl) was analysed by ion pairing HPLC [Gemini C18 column (150×4.6 mm, 5 micron)] with detection at 260 nm using the same separation method described above.

### Isothermal titration calorimetry

All experiments were carried out using a MicroCal VP-ITC calorimeter (MicroCal, Inc.) at 310 K, unless otherwise stated, while stirring at 500 rpm. Experiments were carried out in ITC experimental buffer (50 mM HEPES, pH 8.0, 10 mM MgCl_2_, 0.3 M NaCl, 20% glycerol, 0.15 mM β-mercaptoethanol). *Ft*PPK2 was exchanged into this buffer by dialysis and size exclusion including chromatography. Titrations began with an initial injection of 2 μl followed by 39 identical injections of 5 μl. Data were corrected for heats of dilution by subtracting the data from independent titrations of ligand into buffer. Data were fitted to a bimolecular binding model using MicroCal™ Origin software. Experiments were carried out in duplicate.

### ^31^P-NMR *Ft*PPK2 activity assays

The overall reaction time course of an *Ft*PPK2 catalysed reaction was monitored with ^31^P-NMR. Using standards of ADP and polyphosphate, the relaxation time (T_1_) was optimized to ensure integrals derived from spectra accurately reflected the concentrations of all ^31^P species in the reaction mixture. The signal from nt dCMP (2 mM) was used as an internal standard. The assay mixture (2.5 ml) contained 50 mM Tris/HCl, pH 8.0, 0.3 M NaCl, 20% glycerol, 10 mM MgCl_2_, 80 mM (NH_4_)_2_SO_4_, 10% ^2^H_2_O, 500 μM polyphosphate and up to 2 mM nt substrate. The reaction was initiated through the addition of 150 nM *Ft*PPK2, mixed and data collected at 37°C for 543 s followed by 453-s bins for the duration of the assay. Peak integrals were converted into concentrations using the dCMP standard as a calibrant and the concentration data used to plot time courses, which were fitted to an empirically selected function, either a first order or a linear process.

### *Ft*PPK2 crystallization and structure determination

Initial crystallization conditions were identified using the Hampton Research crystal screen 1 using the sitting drop vapour diffusion method. Conditions for crystallization of *Ft*PPK2 were optimized in 24-well format by the hanging drop vapour diffusion method at 20°C. For X-ray data collection, *Ft*PPK2 was crystallized using a precipitant solution containing: 0.8 M Na citrate, 0.1 M Na HEPES (pH 8.5), 2.5 mM AMP–PNP and 1 mM MgCl_2_. Diffraction data were collected on the i02 beamline at the Diamond Light Source. The data were processed with xia2 [33] and the structure was solved by molecular replacement with the BALBES software [34], which also incorporates the Arp/Warp software [35]. The model was built with COOT [36] and refined with Phenix refine [37]. The data collection and refinement statistics are shown in Table 2.

### Antibiotic sensitivity testing of the ΔFTT1564 *F. tularensis* mutant

All work with *Francisella* strains was performed in a containment level III laboratory in accordance with relevant legislative requirements The *F. tularensis* SCHU S4ΔFTT1564::CAM mutant [17] was tested for susceptibility to various classes of antibiotics. *F. tularensis* SCHU S4 and the ΔFTT1564 mutant strain, were inoculated from a fresh blood cysteine glucose agar (BCGA) plate into 25 ml of brain heart infusion broth to *D*_600_ of 0.1, then the cultures were incubated overnight with shaking at 37°C. Cultures were adjusted to a *D*_600_ of 1.0 with fresh culture medium. In three biological replicates, aliquots of 1 ml of *F. tularensis* SCHU S4 or the ΔFTT1564 mutant were pipetted on to dry BCGA plates and surplus media removed. Sterile discs (BBL™ Sensi-Disc™ Susceptibility Test Discs) 5 mm in diameter, impregnated with an antibiotic, were placed in triplicate on the plate using sterile forceps. The total quantities of antibiotic on each disc were: streptomycin, 10 μg; gentamycin, 10 μg; tetracycline, 30 μ; doxycycline, 30 μg; ciprofloxacin, 5 μg and polymyxin B, 100 μg. The plates were incubated face-up, for 24 h at 37°C and zones of inhibition in the lawns surrounding the discs measured. The mean results from three independent experiments, conducted in technical triplicates were analysed using an unpaired *t*test with Welch's correction for unequal variance using GraphPad Prism V6.02.

## RESULTS

### Expression and purification of *Ft*PPK2

**Figure 2:**
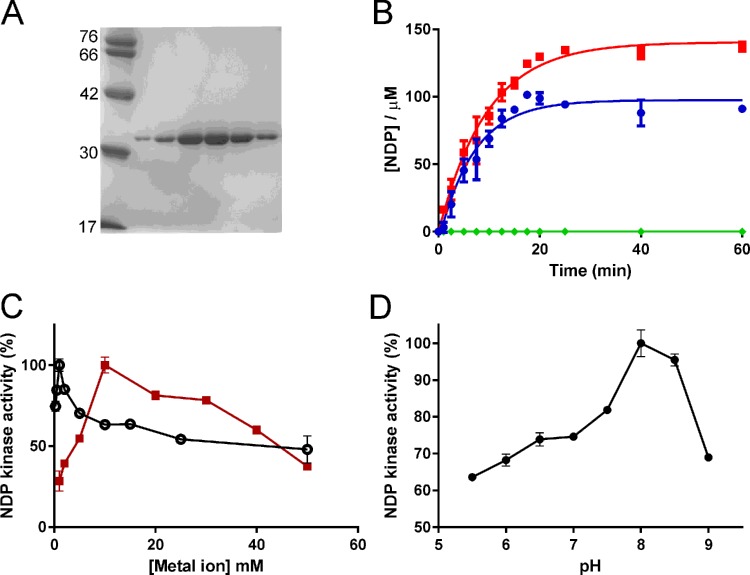
Characterization of *Ft*PPK2 (**A**) SDS/PAGE analysis of fractions from the final purification step (Superdex 75 size exclusion chromatography). (**B**) Formation of NTPs in *Ft*PPK2 activity assays. Concentrations of NTPs were measured by IP–HPLC. Red squares: ATP; blue circles: GTP; green diamonds: UTP. Reagents were added at the following initial conditions: polyphosphate: 0.1 mM; *Ft*PPK2: 250 nM; NDPs: 500 μM; 37°C. (**C**) Metal ion dependence of *Ft*PPK2. Black open circles: Mg^2+^; red squares: Mn^2+^. (**D**) pH dependence of *Ft*PPK2. Activity in (**C**) and (**D**) has been normalized to the maximum (100%).

Heterologous expression *Ft*PPK2 in *E. coli* was greatly improved by using the BL21 Rosetta pLysS (DE3) strain to overcome the problem of codon bias and the A + T rich nature of the *F. tularensis* sequence (66%). Affinity purification with the incorporated His_6_-tag yielded 6 mg of purified *Ft*PPK2/g cell paste and *Ft*PPK2 was stable (over 3 months) when stored as aliquots in buffer containing 20% glycerol at–80°C. Further purification by size exclusion chromatography (Superdex 75) yielded highly purified *Ft*PPK2 suitable for protein crystallization studies (Figure 2A).

### Substrate specificity of *Ft*PPK2

To investigate the substrate preference of *Ft*PPK2, competition activity assays were prepared containing pairs of nucleoside diphosphate analogues (ADP and GDP; ADP and CDP; ADP and UDP) and analysed for formation of the triphosphate product by ion pairing HPLC (Figure 2B). The initial rate was 0.250±0.052 μmol/s for formation of ATP as product and 0.244±0.026 μmol/s for formation of GTP, but UDP and CDP were not substrates under these conditions. Single time point activity assays (quenched at 60 min) were prepared with AMP and GMP (500 μM) as substrates. No formation of nt diphosphates was detected, which demonstrated that AMP and GMP were not substrates for *Ft*PPK2 (result not shown).

### Metal ion and pH dependence of *Ft*PPK2

The optimal concentrations for divalent cations Mg^2+^ and Mn^2+^ with *Ft*PPK2 were 10 and 1 mM respectively (Figure 2C). A lower optimal concentration for Mn^2+^ ions than Mg^2+^ ions has been observed for other PPK2 enzymes [18,23]. *Ft*PPK2 was active over a wide range of pH values, with optimal activity at pH 8 (Figure 2D). The sharp drop-off in activity at pH 9 may have resulted from deprotonation of either a catalytic residue or a protonated basic residue that acts as a counter ion to the polyphosphate and nt substrates.

### Nt substrate steady state kinetics

**Figure 3:**
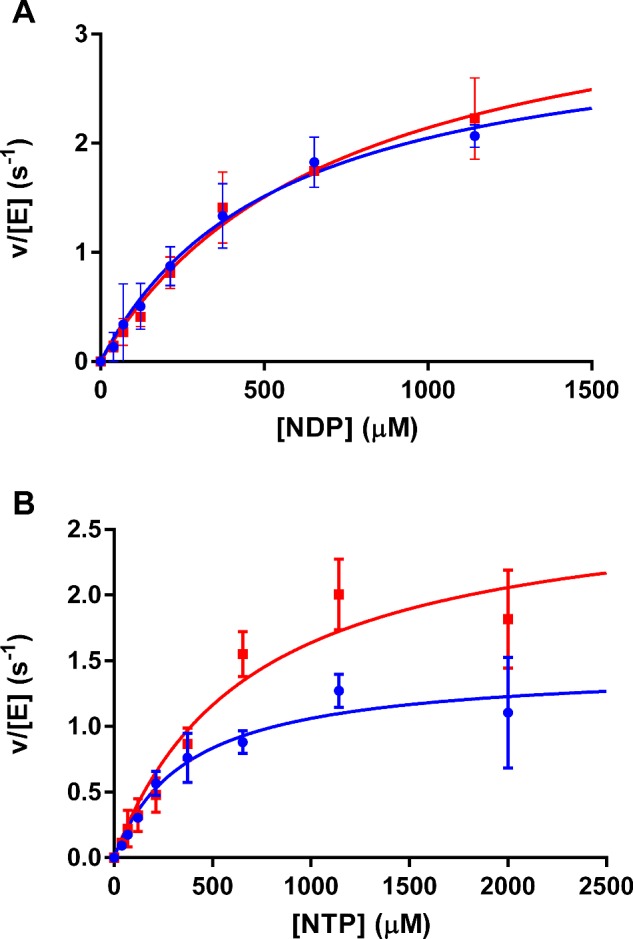
Steady state kinetic analysis of *Ft*PPK2 substrate specificity (**A**) Nucleoside diphosphate substrates, (**B**) nucleoside triphosphate substrates. Adenosine nts: blue circles; guanosine nts: red squares. Reagents were added at the following concentrations: polyphosphate: 0.1 mM; *Ft*PPK2: 150 nM. Goodness of fit (*R^2^*) for each nt was: GDP: 0.99; ADP: 0.99; GTP: 0.95; ATP: 0.97.

As shown in the previous section, *Ft*PPK2 uses both GDP and ADP as substrates and the data indicated that both these substrates were used with approximately equal efficiency. The IP–HPLC assay was then used to quantify (in duplicate) the rates of formation of reaction products in *Ft*PPK2 catalysed reactions, in both directions. For the triphosphate forming reaction, GDP and ADP were used as substrates and the formation of GTP and ATP products were measured. For the reaction in the reverse direction (forming diphosphates), GTP and ATP were used as substrates and the formation of GDP and ADP products were measured. Measurements were made with substrate nts (GDP, ADP, GTP and ATP) concentrations over a range 0–2 mM at 37°C. Fitting the derived initial rates to Michalis–Menten steady state kinetics (Figure 3) gave the *k*_cat_ and *K*_M_ parameters for *Ft*PPK2 which are compared with values for other members of the PPK2 family [23] in Table 1. In the activity assays with nucleoside triphosphates as substrates (Figure 3B), a fine precipitate was occasionally observed to form in assays at higher NTP concentrations (>500 μM). This may account for the observed larger error bars for these activity measurements. The calculated catalytic efficiency (*k*_cat_/*K*_M_) is broadly similar for guanine and adenine nts and for bis- or triphosphate substrates.

**Table 1:** Steady state kinetic parameters for *Ft*PPK2 from activity measurements using IP–HPLC. *Data for *S. meliloti* PPK2 (SMc02148) from Nocek et al.[23].

Substrate	Measured product	*k*_cat_/s^−1^	*K*_M_/μM	*k*_cat_/*K*_M_/s^−1^·M^−1^
ADP	ATP	3.17±0.22	546±79.2	5788±1242
ATP	ADP	1.46±0.13	372±90.5	3920±1301
GDP	GTP	3.69±0.37	727±138	5075±1472
GTP	GDP	2.77±0.41	692±235	4002±1951
*ADP		7.60±0.01	32.0±4.10	23800
*GDP		0.80±0.03	520±70.0	1538

### Substrate binding to *Ft*PPK2 by isothermal titration calorimetry

**Figure 4:**
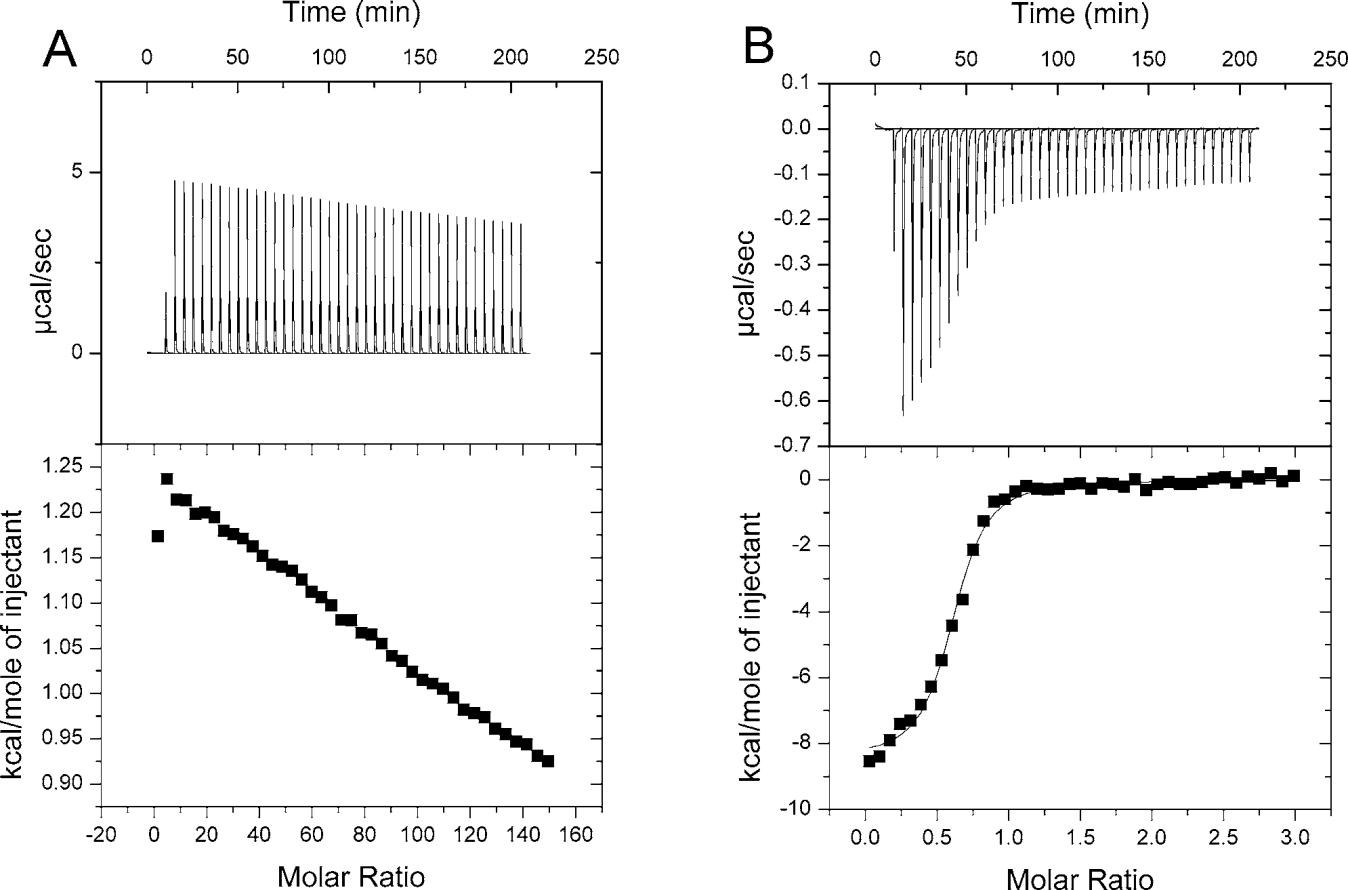
ITC analysis of *Ft*PPK2 substrate binding (**A**) Titration of ADP into *Ft*PPK2. (**B**) Titration of polyphosphate into *Ft*PPK2. Titration conditions: 50 mM HEPES (pH 8), 10 mM MgCl_2_, 0.3 M NaCl, 20% (v/v) glycerol, 0.15 mM β-mercaptoethanol, 298 K.

To measure the binding affinity of substrates to *Ft*PPK2, we used ITC. The results of these titration experiments (Figure 4) showed no discernible substrate binding for a nt substrate in the absence of polyphosphate, but titration of polyphosphate in the absence of nts fitted to a single binding site model (*N*=0.63±0.01) with the following thermodynamic parameters: *K*_a_=8.21±3.29×10^6^, Δ*H*=–6.09±0.17 kcal/mol (1 kcal ≡ 4184 J) and Δ*S*=11.2, equivalent to a *K*_d_ of 122 nM. This data suggest that significant binding of the nt substrate requires the presence of polyphosphate.

### ^31^P-NMR *Ft*PPK2 activity assay

**Figure 5:**
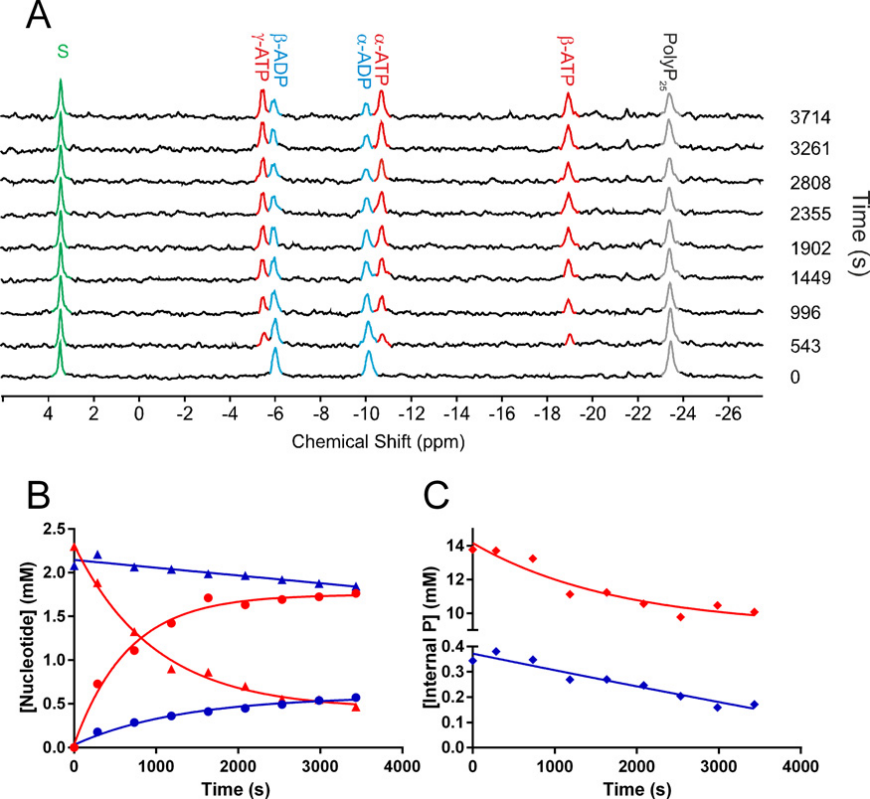
Kinetic analysis of *Ft*PPK2 catalysed reactions using ^31^P-NMR (**A**) Time course for a *Ft*PPK2 reaction monitored by ^31^P-NMR. In this example reaction, initial conditions were: 2 mM ADP, 125 μM polyphosphate, 2 mM dCMP and 150 nM *Ft*PPK2. Peaks were assigned as follows: 3.44 (dCMP),–5.44 (γ-ATP),–5.94 (β-ADP),–10.07 (α-ADP),–10.72 (α-ATP),–18.96 (β-ATP),–23.39 (polyphosphate internal phosphoanhydride); peaks are coloured as follows: ATP: red; ADP: blue; polyphosphate internal phosphoanhydride: grey; dCMP: green. (**B**) Kinetics analysis of ^31^P-NMR time course experiments for two nt concentrations. Experiment 1 (initial [polyphosphate]=500 μM); ATP: red circles, ADP: red triangles. Experiment 2 (initial [polyphosphate]=16 μM); ATP: blue circles, ADP: blue triangles. (**C**) Kinetic analysis of ^31^P-NMR time course experiments for changes in internal phosphoanhydride in polyphosphate. Red: experiment 1; blue: experiment 2. The spectrometer (Bruker 400 MHz, operating at 161 MHz for ^31^P-NMR) was maintained at 37°C for the duration of the experiment.

The overall reaction time course of an *Ft*PPK2 catalysed reaction was monitored with ^31^P-NMR (Figure 5). Using standards of ADP and polyphosphate, the relaxation time (T_1_) was optimized to ensure integrals derived from spectra accurately reflected the concentrations of all ^31^P species in the reaction mixture. The signal from nt dCMP (2 mM) was well separated from any substrate- or product-derived signals and was used as an internal standard (Figure 5A, green). The assay mixture (2.5 ml) contained 50 mM Tris/HCl (pH 8.0), 0.3 M NaCl, 20% glycerol, 10 mM MgCl_2_, 80 mM (NH_4_)_2_SO_4_, 10% ^2^H_2_O, up to 500 μM polyphosphate and up to 2 mM nt substrate. The reactions were initiated through the addition of 150 nM *Ft*PPK2, mixed and data collected at 37°C for 543 s followed by 453-s bins for the duration of the assay. Qualitatively, the time-dependent utilization of the ADP [signals at–5.94 (β-ADP) and–10.07 (α-ADP) ppm] and the internal phospho-anhydrides of polyphosphate (–23.39 ppm) can be observed, as can the corresponding formation of ATP [signals at–5.44 (γ-ATP)–10.72 (α-ATP);–18.96 (β-ATP) ppm]. No signal for the formation of P_i_ or other phosphorus containing by-product appeared, suggesting the *Ft*PPK2 catalysed phosphotransfer reaction is efficient, without any significant competing hydrolytic or other side reaction. Using the dCMP standard as an internal calibrant, the integrals were converted into concentrations for each time point. Plotting these concentrations against time gave a reaction time course for ATP, ADP (Figure 5B) and polyphosphate internal phosphoanhydride (Figure 5C). Comparing time courses initiated at high and low polyphosphate concentrations (500 and 16 μM respectively) reveals the limitation of this experimental approach, as the errors in the integrals at lower polyphosphate concentrations become too large. For example, using 500 μM polyphosphate (Table 2, experiment 1), the kinetic parameters for fitting the time courses of ATP formation and ADP depletion approximately match, as does the total change in concentrations and the calculated initial rates. However, the results at 16 μM polyphosphate, while qualitatively heading in the expected direction, cannot be fitted to give well correlated results. A similar caveat must be placed on integrals of the internal phosphate signal of polyphosphate, which both qualitatively show a decrease but the small change in a relatively large integral could not be reliably fitted (Figure 5C).

**Table 2:** Rate constants from ^31^P-NMR time courses of *Ft*PPK2 assays

			Nts	
Experiment*	Initial [PolyP] /μM	Kinetic parameters^†^	ATP	ADP
1	500	*k*_1_ × 10^−3^/s^−1^	1.52±0.17	1.02±0.14
		*R*^2^	0.99	0.99
		ΔC/*μ*M	1750±73	1880±36
		Initial rate/*μ*M·s^−1^	2.65±0.41	1.915±0.30
2	16	*k*_1_ × 10^−3^/ s^−1^	0.769	ND^‡^
		*R*^2^	0.98	ND
		Δ*C*/*μ*M	556	ND
		Initial rate/*μ*M·s^−1^	0.450	ND

*Reactions also contained 150 nM *Ft*PPK2, 2 mM nt, 2 mM CMP (internal standard) and 10% v/v ^2^H_2_O.

†Time courses were fitted to a first order process: *k*_1_, first order rate constant; *R*^2^, goodness of fit; Δ*C*, calculated change in concentration of nt.

‡ND, not determined (a reliable fit could not be achieved).

### X-ray crystal structure of *Ft*PPK2

**Figure 6:**
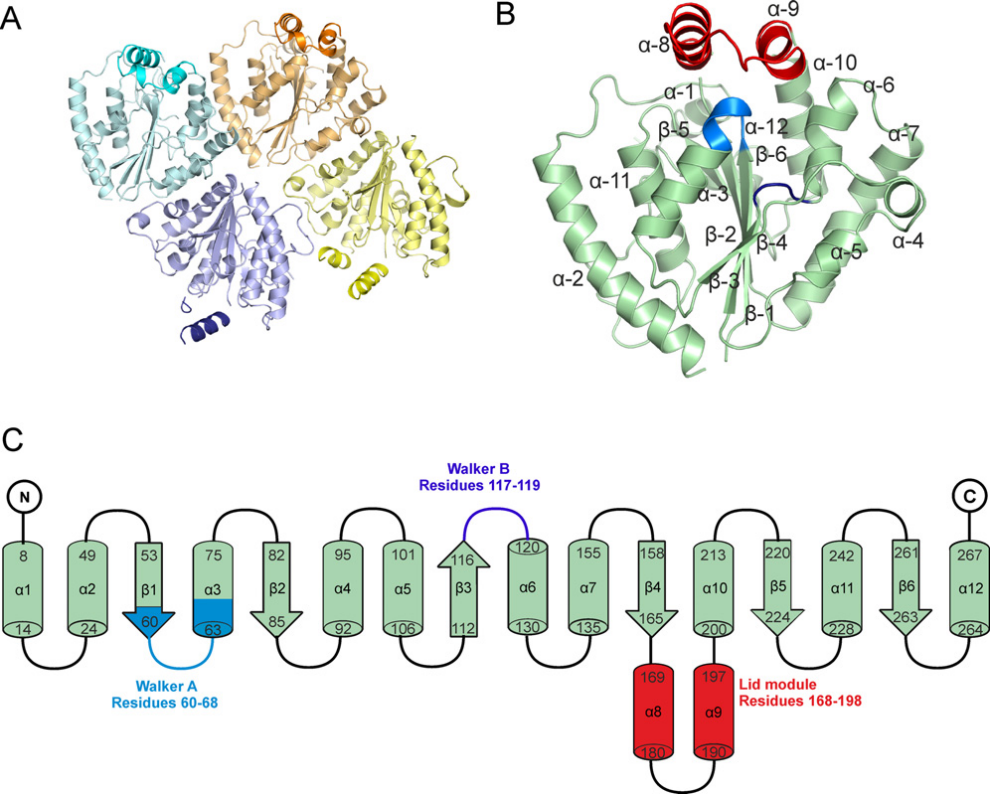
Overall structure of *Ft*PPK2 (**A**) Tetrameric organization of *Ft*PPK2 within the asymmetric unit. (**B**) *Ft*PPK2 monomer A, labelling the α-helices (α-1–12) and strands of β-sheet (β-1–6). The lid-loop motif, including helices α-8 and α-9, is coloured red, the Walker A and B motifs are coloured blue and purple respectively. (**C**) Topology diagram colour coded as in (B).

Screening using the sitting drop vapour diffusion method yielded *Ft*PPK2 crystals with a sodium citrate precipitant and HEPES buffer (pH 8.4) in the presence of the non-hydrolysable ATP analogue adenylyl imidodiphosphate (AMP–PNP) [38]. The crystals formed overnight and reached a maximum size after 3 days. Conditions were optimized to produce crystals up to 200 μm in the longest dimension which diffracted to a 2.23 Å (1 Å=0.1 nm) resolution at Diamond beamline i02 (Table 3). The *Ft*PPK2 structure was solved by molecular replacement using the *Sinorhizobium meliloti* PPK2 structure (SMc02148, PDB ID: 3CZQ) as a model and the BALBES pipeline [34]. The refined structure of *Ft*PPK2 reveals four monomers in the asymmetric unit in a D2 tetrameric organization (Figure 6A). Despite the inclusion of AMP–PNP in the crystallization solution, no density corresponding to this ligand was observed. Each monomer consists of a six-strand β-sheet, surrounded by 10 α-helices, with the insertion of a lid motif (helices 8 and 9; Figures 6B and 6C). A Dali search [39] for structural similarity identified PPK2 from *S. meliloti* as the closest related protein structure, followed by the PPK2 protein from *Pseudomonas aeruginosa* (PA3455, PDB ID: 3CZP) [23] and *Arthrobacter aurescens* (AAur_2811, PDB ID: 3RHF). The next most similar structures are two thymidylate kinases, from *Sulfolobus tokodaii* STK_15430, PDB ID: 2PLR) and *Staphylococcus aureus* (SAV0482, PDB ID: 4EAQ) respectively (Table 4).

**Table 3:** Crystallographic data for *Ft*PPK2. Figures in brackets indicate the highest resolution shell.

Data Set	*Ft*PPK08
Resolution of data (outer shell; Å)	81.65–2.23 (2.27–2.23)
Space group	P2_1_2_1_2_1_
Unit cell parameters	a=86.79, b=88.89, c=163.3 a=b=c=90°
*R*_merge_ (overall, all I+ and I–)	0.052 (0.539)
Mean I/σI (outer shell)	15.5 (2.4)
Completeness (outer shell; %)	99.2 (99.7)
Multiplicity (outer shell)	3.6 (3.5)
No. unique reflections	61793
*R*_work_	0.20
*R*_free_	0.25
Number protein atoms	8412
Number solvent waters	473
Number ligand atoms	None
RMSD for bonds (Å)	0.009
RMSD for angles (°)	1.220
Average protein B factor	31.6
Average lid module B factor	45.8
Average solvent B factor	48.8
PDB entry	4YEG

**Table 4:** Dali analysis for FtPPK2. Results for the five structures that are most similar to *Ft*PPK2 are shown.

Model PDB code	Average Z-score	Average RMSD (Å)	Sequence Identity (%)
3CZQ	31.78	1.73	47.0
3CZP	24.65	2.25	33.0
3RHF	24.75	2.53	26.8
4NZY	11.65	3.2	29.5
2PLR	11.6	3.25	18.5

**Figure 7:**
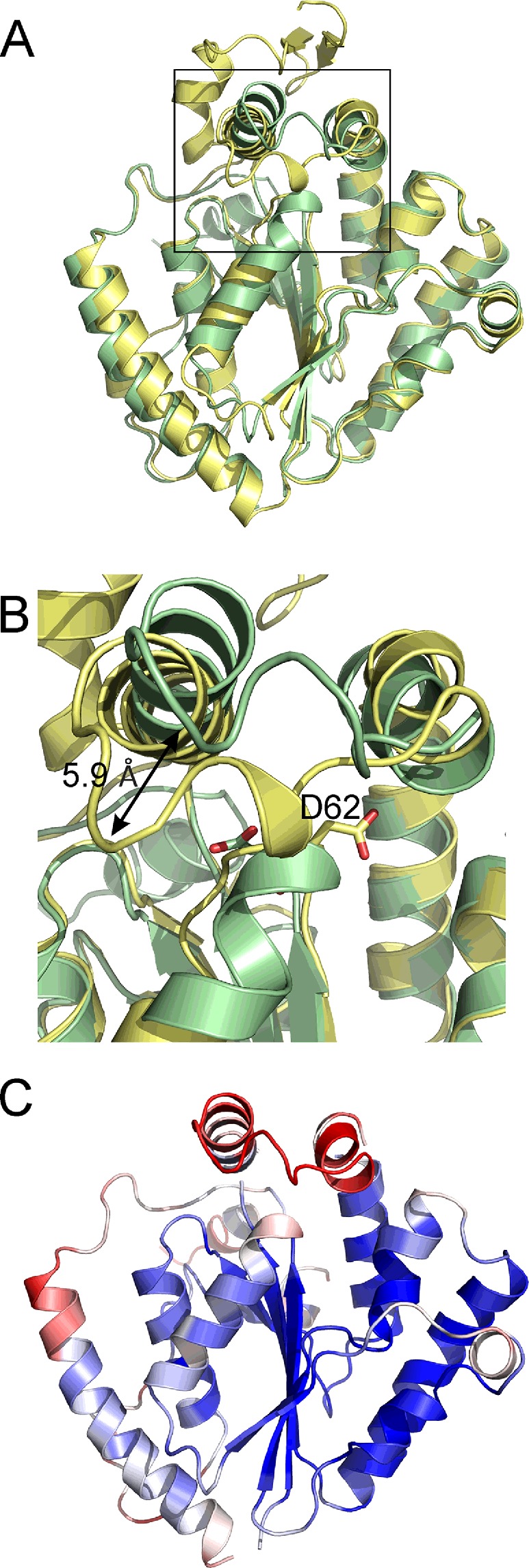
Similar and flexible regions of PPK2 (**A**) Overlay of the *F. tularensis* (pale green, PDB: 4YEG) and *S. meliloti* (pale yellow, PDB: 3CZQ) PPK2 structures. (**B**) Detail of the active site region [boxed region in (A)] highlighting the movement in the lid module (up to 5.9 Å) and the movement of the Walker A motif aspartic acid residue (*Ft*PPK2 D62 and *Sm*Ppk D93). (**C**) Structure of *Ft*PPK2 coloured by B-factors, ranging from blue (20 Å^2^) to red (50 Å^2^).

Comparison of *Ft*PPK2 and *S. meliloti* PPK2 (Figure 7A) shows high structural similarity in most areas (RMSD 0.782 Å for all atoms), apart from the N-terminus, Walker A motif and lid module. Like the *S. meliloti* structure, *Ft*PPK2 has a central six-stranded parallel β-sheet flanked by α-helices at the side and top. The lid module of *Ft*PPK2 is not covered by the N-terminal extension domain present in the *S. meliloti* structure and the *Ft*PPK2 lid module appears to be in a slightly more open conformation (Figure 7B). Parts of the electron density for the lid module for fully refined *Ft*PPK2 cannot be resolved for chains C (missing residues 181–192) and D chains (missing residues 182–188), which may point to a high degree of flexibility in this region. This flexibility may also be reflected in the lid B factors which are higher in the lid motif (for example, residues 168–198, chain A, mean B=45.8 Å^2^) than the mean for the whole structure (all residues, chain A, mean B=31.6 Å^2^; Figure 7C).

**Figure 8:**
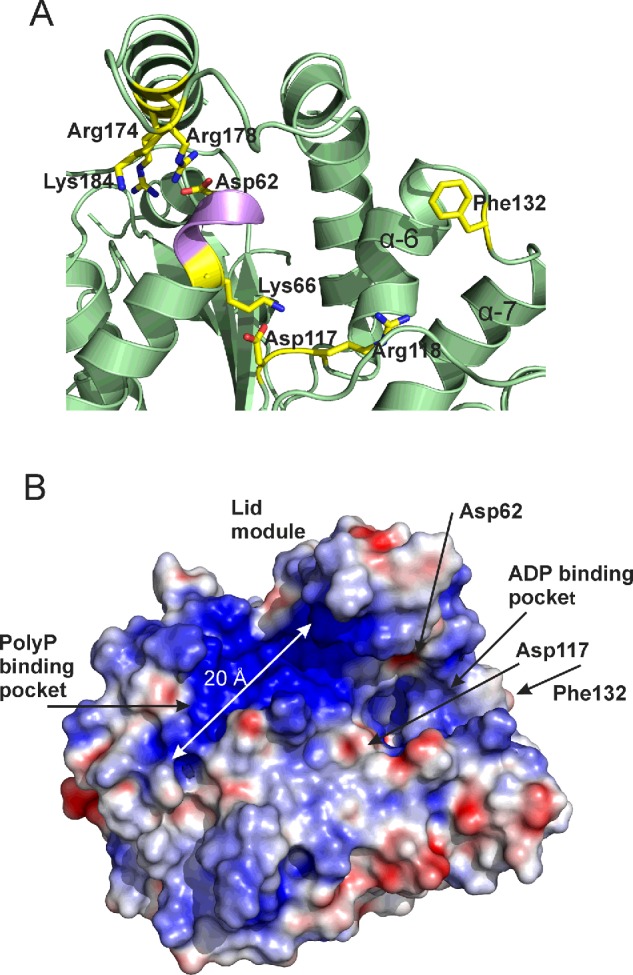
Active site of *Ft*PPK2 (**A**) Residues close to the active site with proposed roles in substrate binding or catalysis. (**B**) *Ft*PPK2 with an electrostatic surface, showing proposed substrate-binding sites and the location of residues with proposed roles in catalysis. Electrostatic map generated with APBS [50] using a–20 to +20 scale.

Nocek et al. [23] postulated that the nt substrate binds to PPK2 to one side of the Walker A-motif containing loop, which in *Ft*PPK2 corresponds approximately to the area between Asp^117^ and Phe^132^, forming a pocket between α helices 6 and 7 (Figures 6 and 8A). They suggested that a conserved aspartic acid residue (corresponding to *Ft*PPK2 Asp^117^; Figure 8A) in the Walker B motif may co-ordinate a magnesium ion required for substrate binding and potentially for catalysis. In addition they identified a conserved lysine residue in the Walker A motif (corresponding to *Ft*PPK2 Lys^66^; Figure 8A) which they anticipated may bind the β- and γ-phosphates of ATP in the active site.

*Ft*PPK2 has a large positively charged region (on the left hand side of the Walker A motif as shown in Figure 8B), made up of residues His^76^, Arg^174^, Lys^228^, Lys^229^ and Arg^232^. We propose these residues constitute the polyP substrate-binding pocket, which is sufficiently long (18 Å) to accommodate approximately eight residues of the polyphosphate chain (calculated maximum length 21 Å). Using this arrangement, the terminal phosphate of the polyP and the nt are correctly juxtaposed below the lid, in the region of the Walker A motif and the catalytically important Lys^66^ and Asp^62^. Higher B-factors indicate that the Walker A motif is a flexible region and the N-terminal region of helix 3, around Asp^62^ in the *Ft*PPK2 structure, adopts a different conformation to the *S. meliloti* PPK2 structure, with some atoms moving as much as 5.9 Å (Figure 7B), although the functional significance of this movement is difficult to ascertain without precise knowledge of the substrate-binding modes. There are several conserved basic residues in the lid module that are close enough to interact with the poylphosphate ligand: Arg^178^, Arg^174^ and Lys^184^. The two arginine residues, Arg^174^ and Arg^178^, form part of a motif (R-X_2–3_-R) conserved in the lid of bacterial PPK2s [40]. The conserved Arg^118^ of the Walker B motif is anticipated to potentially form hydrogen bonds with the β- and γ-phosphates of the nt substrate.

### Antibiotic sensitivity of *F. tularensis* ΔFTT1564 mutant

**Figure 9:**
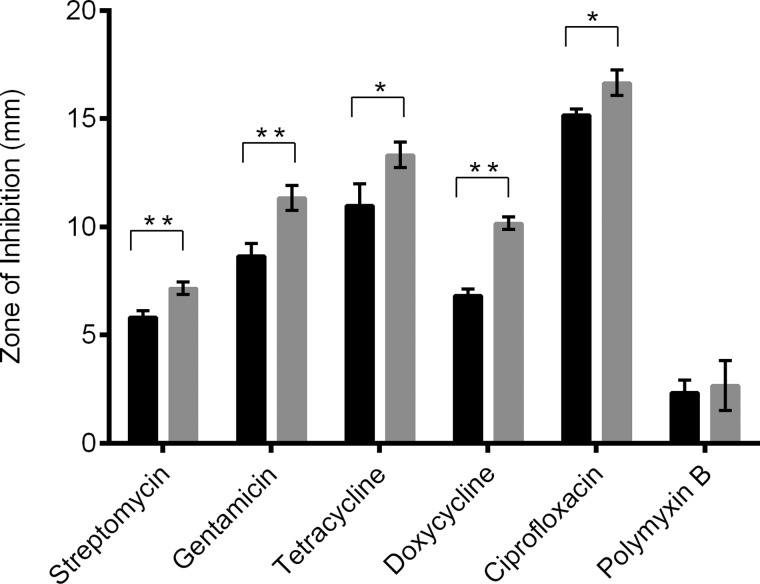
Antibiotic sensitivity of *F. tularensis* SCHU S4 wild-type and mutant strain ΔFTT1564 Zones of inhibition were measured in bacterial lawns surrounding antibiotic impregnated discs. Black bar: wild-type; grey bar: ΔFTT1564 mutant. Statistical significance determined by unpaired *t*test with Welch's correction for unequal variance (*P ≤ 0.05, **P ≤ 0.01).

The effect of inactivation of polyphosphate production in *F. tularensis* on antibiotic susceptibility was determined (Figure 9). Relative to the wild-type strain, the ΔFTT1564 mutant was significantly more sensitive to killing by antibiotics targeting the translational machinery [41], namely streptomycin (10 μg, *P*=0.0048), gentamicin (10 μg, *P*=0.0048), tetracycline (30 μg, *P*=0.0357) and doxycycline (30 μg, *P*=0.0028). The ΔFTT1564 *F. tularensis* mutant was also more susceptible to killing by the topoisomerase/gyrase inhibitor ciprofloxacin, (5 μg, *P*=0.0286). However, the mutant showed no difference in susceptibility to the membrane-targeting compound polymyxin B.

## DISCUSSION

The characterization of *Ft*PPK2 through the HPLC assay format has enabled measurement of substrate preference and specificity (Figure 2; Table 1), identifying that *F. tularensis* polyphosphate kinase belongs to the PPK2 family [18,23,24,42]. *Ft*PPK2 did not accept pyrimidine nt or purine monophosphate substrates, but comparing activity with the purine substrates, it showed little substrate preference between guanosine and adenosine nts. This is comparable with the observed small preference of *M. tuberculosis* PPK2 [18] for ATP (*K*_M_=330 μM) over GTP (*K*_M_=660 μM), but very different from *S. meliloti* PPK2 [23] which shows a 10-fold difference in *K*_M_, preferentially utilizing ADP over GDP (Table 2).

Sequence analysis has indicated that polyphosphate kinase enzymes are widely distributed in prokaryotes [8]. Some bacteria have a single polyphosphate kinase, either of the PPK1 or of the PPK2 subtypes and other species of bacteria contain multiple polyphosphate kinase sequences, including a mixture of subtypes [8]. Analysis of the genomes of *F. tularensis* subspecies *tularensis* SCHU S4 and subspecies *novacida* indicates that genes FTN1472 and FTT1564 respectively encode a member of the PPK2 family [17]. Moreover, biochemical evidence from the knockout mutant verified this to be the only gene encoding a polyphosphate kinase [17]. *Francisella* spp*.* are renowned for having compact genomes [43] and therefore there may be a competitive advantage to avoiding having multiple polyphosphate kinases when one broader specificity enzyme will do.

Measuring the formation of a product nt by HPLC has a significant limitation, as the sensitivity of absorbance at 260 nm limits accurate measurement of nts from assay mixtures to approximately 5 μM; below this point, the errors become unacceptable. This was not an issue for the nt substrates (*K*_M_’s in the range 300–800 μM), but the *K*_M_ for polyphosphate was significantly lower and could not be accurately measured with the HPLC-based assay. Using ITC (Figure 4), the binding of polyphosphate could be quantified for a single binding site and gave a sub-micromolar binding constant. This contrasted with the lack of nt binding observed in the absence of polyphosphate. Further characterization of *Ft*PPK2 with respect to polyphosphate turnover was achieved using ^31^P-NMR analysis. On a qualitative basis, the ^31^P-NMR assays confirmed the utilization of polyphosphate and ADP as substrates and the formation of ATP. The limited sensitivity of the ^31^P-NMR restricted quantitative measurements of activity to assays containing relatively high concentrations of substrates (initial [NDP]=2 mM; initial [polyphosphate]=500 μM): under these conditions, there was a good correlation of ATP formed and ADP utilized and the associated rate constants (Table 2, initial [polyphosphate]=500 μM). Interestingly, the fidelity of the phosphotransfer reaction proved to be excellent: over the relatively long time course of these experiments (over 1 h corresponding to more than 10000 turnovers), by-products of hydrolysis such as P_i_ were not observed to accumulate to a measurable extent.

There is a high degree of similarity between the structures of *Ft*PPK2 and *S. meliloti* PPK2 enzymes (Figure 7A), although the *Ft*PPK2 sequence is shorter than the other structurally characterized PPK2 enzymes and lacks the N-terminal domain present in the *P. aeruginosa* sequence (up to residue ∼60 of PA0141, accession code UniProtKB Q8GCQ3) [25]**.** In terms of active site binding, ITC identified that *Ft*PPK2 does not significantly bind nt substrates in the absence of polyphosphate, which may explain the lack of observable ligand density for a nt in the structure of a crystal grown in the presence of AMP–PNP. Analysis of the surface electrostatic potential has identified an extensive positively charged region suitable for polyphosphate binding. Nocek et al. [23] proposed NDP/NTP binding between helices 6 and 7, but the molecular basis for substrate selectivity (purine compared with pyrimidine, guanosine compared with adenosine) and assignment of precise roles for active site residues during catalysis will require further experimental evidence, either from mutagenesis or from structural analysis of *Ft*PPK2 co-crystallized with substrate(s) or structural analogue(s). It seems likely that residues in the lid module are involved in substrate binding [23] and it will be interesting to determine if there is the same flexibility in the lid module in a substrate-bound *Ft*PPK2 structure or not.

As polyphosphate appears to be an important metabolite contributing to survival of *F. tularensis*, it was important to ensure that abolition of polyphosphate production did not inadvertently induce a stress response that made the pathogen more resistant to clinically relevant antibiotics. Disc diffusion experiments indicated that decreased polyphosphate production in *F. tularensis* led to increased antibiotic sensitivity to various classes of anti-microbials. An important caveat must be applied to these experiments, as a chromosomal deletion can result in polar effects, modifying the expression of downstream genes. An approach that may form part of future studies of antibiotic sensitivity is provided by complementation analysis of the ΔFTT1564 strain, which has potential to address this issue. Ciprofloxacin is the current antibiotic therapy of choice, according to the CDC guidelines, for the treatment of tularemia and has very effective antibiotic action against all *F. tularensis* strains [44]. Crucially, the abolition of polyphosphate production resulted in increased susceptibility to ciprofloxacin. This suggests that inhibitors of *Ft*PPK2 could act as ‘antibiotic adjuvants’ to enhance the efficacy of current antibiotic regimens for the treatment of tularemia, as well as acting as antibiotics in their own right. In contrast, the ΔFTT1564 mutant showed no statistically significant increase in sensitivity to polymixin B. Polymyxin B disrupts the bacterial cell membrane [45] and *F. tularensis* has known resistance; resistance is conferred by a tetra-acylated lipid A to which polymyxin B cannot bind [46]. Therefore, inactivation of polyphosphate production does not render the pathogen generally weaker due to stress, but rather the increased susceptibility is due to a specific inability to counter antibiotic killing mechanisms.

Previous studies into other Gram negative bacteria such as *P. aeruginosa*, have reported increased susceptibility to antibiotics in mutants that are defective for genes involved in the stringent response [47]. Polyphosphate production is directly influenced by the stringent response whereby abolishment of (p)ppGpp synthesis results in simultaneous decreased polyphosphate production [48]. It has also been reported that bacteria become more tolerant to antibiotics under nutrient starvation conditions [49]. That targeted mutagenesis of FTT1564 not only results in decreased polyphosphate production [17] but also in an increase in sensitivity to antibiotics, indicates that polyphosphate metabolism plays a key role in the increased antibiotic susceptibility observed upon inactivation of the stringent response.

In summary, we report the biochemical and biophysical characterization of *Ft*PPK2, an enzyme activity important for *F. tularensis* virulence [17]. The enzyme can serve as a broad specificity reversible purine diphosphate kinase, consistent with *Ft*PPK2 being the only polyphosphate kinase encoded by the *F. tularensis* genome. Inhibitors of *Ft*PPK2 could be novel antibiotics and may also enhance the activity of other antibiotics. The combined biochemical, biophysical and microbiological results reported herein, addresses some of the prerequisites for studies to discover such novel *Ft*PPK2 inhibitors.

## ACCESSION CODES

The co-ordinates for *Ft*PPK2 have been deposited in the PDB with the PDB ID 4YEG.

## Abbreviations

AMP-PNP: adenylyl-imidodiphosphate
BCGA: blood cysteine glucose agar
CDC: Centers for Disease Control and Prevention
DMHA: dimethylhexylamine
*Ft*: *Francisella tularensis* subspecies *tularensis* SCHU S4
*Ft*PPK2: *F. tularensis* polyphosphate kinase
IP: ion pairing
ITC: isothermal titration calorimetry
MES: 2-(N-morpholino)ethanesulfonic acid
NMP: nucleotide monophosphate
PPK2: polyphosphate kinase 2
Ppx: polyphosphatase
*Sm*: *Sinorhizobium meliloti*
2YT: yeast extract and tryptone media
